# Novel use of local analgesia prior to intramuscular magnesium sulphate injection compared to mixed local analgesia with magnesium sulphate to reduce pain: a randomised crossover study in patients being managed for eclampsia and preeclampsia

**DOI:** 10.3389/fpain.2024.1376608

**Published:** 2024-07-11

**Authors:** Modimowame Jamieson, Rebecca Luckett, G. Justus Hofmeyr

**Affiliations:** ^1^ Department of Obstetrics and Gynaecology, Princess Marina Hospital, Gaborone, Botswana; ^2^ Department of Obstetrics and Gynaecology, University of Botswana, Gaborone, Botswana; ^3^ Department of Obstetrics and Gynaecology, Botswana Harvard AIDS Institute Partnership, Gaborone, Botswana; ^4^ Department of OBGYN, Beth Israel Deaconess Medical Centre, Boston, MA, United States; ^5^ Department of Obstetrics and Gynaecology, University of the Witwatersrand and Walter Sisulu University, East London, South Africa

**Keywords:** intramuscular injection, lignocaine, local anaesthesia, magnesium sulphate, pain

## Abstract

**Objective:**

The World Health Organization (WHO) recommended addition of local anesthetic to reduce the intense pain of intramuscular injection of 50% Magnesium Sulphate (MgSO_4_) salt solution has been found to be ineffective. We tested whether giving the local anesthetic 5 min before the MgSO_4_ injection would reduce pain.

**Methods:**

We conducted a prospective cross-over trial where each participant with pre-eclampsia or eclampsia received sequential and mixed injection methods in random sequence during sequential MgSO_4_ administrations. Pain and preference were assessed using descriptive words, a numeric pain scale and direct comparison between the two injection methods. Differences were measured using the Wilcoxon signed rank test, risk ratios with 95% confidence intervals and the Chi squared or Fisher's test. The administration techniques were refined based on an initial pilot of 8 participants.

**Results:**

We enrolled 49 consented participants and analysed data from 41 post-pilot participants The sequential injection method had a non-significantly lower mean pain score than the mixed injection method (3.1 vs. 3.3, *p* = 0.44). Severe pain was reported for 3/41 vs. 9/41, *p* = 0.12. The sequential injection method was perceived to be more painful by 13 (37%) vs. 22 (63%) participants (*p* = 0.03). The sequential injection was preferred by 21(60%) vs. 14 participants (40%) (*p* = 0.1).

**Conclusion:**

Our results consistently favoured the novel sequential injection method. The lack of statistical significance for most results is not surprising given the small sample size. Given the potential for clinically important benefits to women, a larger study to confirm these results is justified.

**Clinical Trial Registration:**

https://pactr.samrc.ac.za/, Identifier (PACTR202201521544765).

## Introduction

Magnesium sulphate (MgSO_4_) is the international standard-of-care anticonvulsant for the management of eclampsia and preeclampsia (1, 2). MgSO_4_ can be administered by intravenous (IV) and intramuscular (IM) routes, the Zuspan (3) and Prichard (4) regimens respectively. In low and middle-income countries like Botswana, use of the Zuspan (IV) regimen is not common because infusion pumps are not available and continuous monitoring required for manually administered IV administration is not feasible. IM administration of MgSO_4_ is the preferred method given these safety limitations, however, it is given in a large volume (10 ml, 5 g of 50% MgSO_4_) of highly concentrated salt solution and is exceptionally painful (5). Severe and very severe pain with IM injection of MgSO_4_ has been reported by 55% of women, which results in lower compliance for IM MgSO_4_ administration in comparison to IV infusion (6).

The World Health Organisation (WHO) guidelines recommend addition of lignocaine to IM injections of MgSO_4_ (7), yet there are few data to support this practice. One randomized trial found no benefit with the addition of lignocaine to MgSO_4_ injection in reducing pain at the site of injection (mean pain score 5.23 vs. 5.26 for MGSO_4_ alone) (5). Other methods of reducing pain with IM injections which have been studied include lavender inhalations (8), manual acupressure (9) and cryotherapy (10).

This limited size, preliminary proof of concept study aimed to evaluate whether a novel method of giving local analgesia with 2% lignocaine five minutes prior to intramuscular injection of MgSO_4_ (sequential injection method) shows potential to reduce pain at the injection site compared to the current standard administration of mixed 2% lignocaine and MgSO_4_ (mixed injection method), and to assess participant's preference for the injection method.

## Materials and methods

This randomised crossover trial was conducted in Princess Marina Hospital, Gaborone, the largest referral hospital in Botswana, serving the southern part of the country.

Participants were recruited from the labour ward, antenatal clinic, antenatal ward and postnatal ward from 4th May 2022 till 23rd July 2022. The investigator was informed about potential study participants by colleagues when admitting patients with preeclampsia or eclampsia who were to receive or receiving MgSO_4_. The researcher assessed patients for eligibility. Eligibility criteria were: 18–50 years and, diagnosed with preeclampsia or eclampsia, receiving magnesium sulphate, conscious, willing and able to give consent and provide answers to questions about pain and preference. Eligible participants were informed about the study, and both the sequential injection and mixed injection methods and the pain rating scale were explained in their preferred language (either English or Setswana). All questions were answered by the investigator and written informed consent obtained from those willing to participate. After signing the consent form the participants were educated on the numeric pain scale of 0–10 using the participants preferred language (Setswana or English) and each given a numeric pain scale to circle the pain experienced immediately after the injection.

A sample size of 41 was chosen to detect a reduction in the primary outcome (severe pain) from 75% to 45% with 95% certainty and 80% power (Epi Info software).

Participant's demographic information, diagnosis and history of receiving MgSO_4_ was obtained and entered on the case record form. Computer generated randomization was used by a researcher not involved in the clinical work to prepare numbered sealed opaque envelopes indicating which method of injection the participant should receive first. The participant's name was entered on a numbered register and the participant opened the matching numbered sealed envelope.

The procedure for the sequential injection method was refined in a pilot group of the first 8 participants. Initially, for the sequential injection method, different size needles were used for the lignocaine injection and the MgSO_4_ injection and it was found to be difficult to ensure that the MgSO_4_ injection was placed at the precisely the same site as the lignocaine injection. The method was modified to use the same needle, left in position between the lignocaine and the MgSO_4_ injections.

For the sequential injection 1 ml of 2% lignocaine was administered in the upper outer quadrant of either the left or right buttock, using a 21 gauge needle and the needle was left in position. After 5 min, 10 ml of 50% MgSO_4_ (5 g MgSO_4_) was administered through the same needle. For the mixed injection method, 1 ml of 2% lignocaine was mixed with 10 ml of MgSO_4_ in one syringe and the administered as a single injection using a 21 gauge needle on the upper outer quadrant of the buttock. The primary investigator prepared and administered all injections to the participant. The participant then received the alternative injection method when due for the next MgSO_4_ injection (immediately for loading doses, or after 4 h for maintenance doses). This allowed participants to serve as their own controls. In Princess Marina Hospital MgSO_4_ is given as 14 g loading dose, 4 g in 200 ml normal saline IV and 10 g IM as 5 g to each buttock, followed by maintenance 5 g IM every 4 h for 24 h.

Participants were asked to rate the pain at the injection site by indicating the number correlating with the perceived pain on a numeric pain rating scale of 0–10 immediately after receiving the injections. Participants were also asked to choose the word best describing their pain: no pain, mild pain, moderate pain and severe pain. After both administrations, participants were asked to judge which was more painful, and which method of injection they would prefer to receive in the future if they were to receive IM MgSO_4_ again.

The study data was entered onto paper case record forms. The data was entered from the case record form into an Excel spreadsheet and checked for errors. Statistical analysis was conducted using Epi Info 7 and Stata version 17. Mean, standard deviation, minimum and maximum pain scores were computed and compared with the Wilcoxon matched–pairs signed–rank test with 95% confidence intervals. Proportions were compared using the Chi Square test or the Fisher's test for small numbers less than 5. *P*-value less than 0.05 was considered statistically significant.

Institutional review board (IRB) approval was obtained from the University of Botswana (reference UBR/RES/IRB/BIO/GRAD/160, 6 October 2021), Ministry of Health and Wellness Research Unit, and Princess Marina Hospital IRB Committee [reference PMH 2/2A(7)/153, 10 January 2022]. The study was registered with the Pan African Clinical Trials Registry (PACTR) on 13th March 2021, (CLINICAL TRIAL REG Number PACTR202201521544765) assessable on https://pactr.samrc.ac.za/TrialDisplay.aspx?TrialID=15746. The actual recruitment started on 4th May 2022, ended 23rd July 2022 and the clinical trial completion was 23rd July 2023 after the sample size was met.

## Results

**Figure 1:**
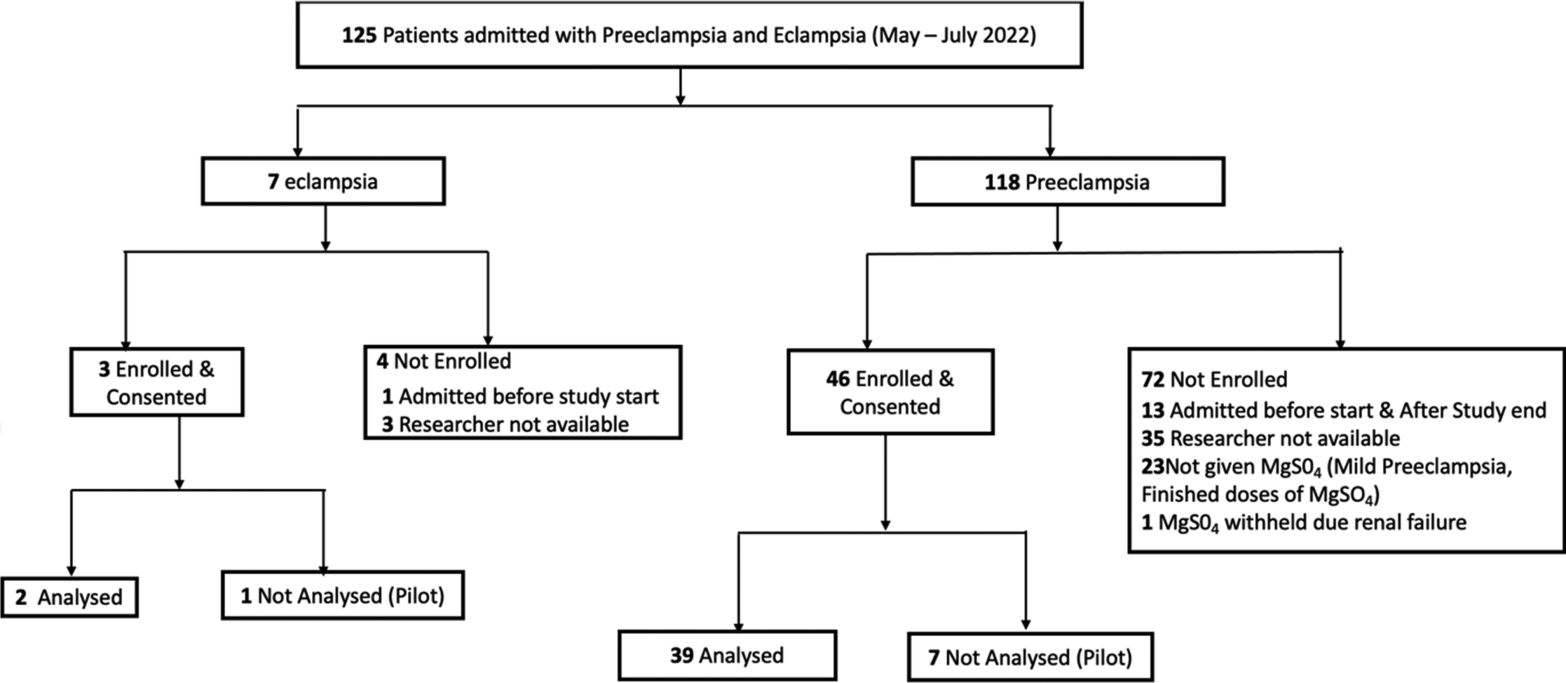
CONSORT flow diagram of patients admitted with pre-eclampsia or eclampsia.

During the study period from 2 May to 29 July 2022, a total of 125 patients were admitted to Princess Marina Hospital with a diagnosis of preeclampsia (118) and eclampsia (7). A total of 49 participants scheduled to receive MgSO_4_ gave written informed consent and were enrolled in the study (see CONSORT flow diagram Figure 1). The first 8 participants constituted the pilot group to refine the sequential method procedure, thus a total of 41 participants were included in the study.

The mean age of the study participants was 31 (range 18–43) years, the mean gravidity was 2.6 (1–5) and mean parity 1.4 (0–4). Most of the study participants (78.1%) had secondary education, 39 (95%) were diagnosed with preeclampsia, and 2 (5%) with eclampsia and 3 (7.32%) had a history of receiving magnesium sulphate in their previous pregnancy. Most of the study participants received the loading dose of MgSO_4_ 28 (68.3%) and 13 (31.7%) received the maintenance doses of MgSO_4_, as shown in Table 1.

**Table 1:** Demographic characteristics of participants.

Characteristics *N* = 41	Mean (standard deviation)	Range
Age (years)	30.5 (6.90)	18–43
Gravidity	2.59 (1.37)	1–5
BMI (kg/m^2^)	31.9 (8.20)	19.42–55.9
Parity	1.39 (1.20)	0–4
	Frequency	Percentage
Nulliparous	12/41	29.3
Education		
Secondary	32/41	78.1
Tertiary	9/41	22.0
Diagnosis		
Preeclampsia	39/41	95.1
Eclampsia	2/41	4.88
Timing of MgSO_4_ administration		
Antenatal	37/41	90.2
Postnatal	4/41	9.76
History of receiving MgSO_4_ in a prior pregnancy	3/41	7.32
MgSO_4_ received		
Loading dose	28/41	68.3
Maintenance dose	13/41	31.7

The pain scores are shown in Table 2. The mean pain score for the sequential injection method was 3.1 (0–9) and for the mixed injection method was 3.3 (0–10). The difference was not statistically significant (*p* = 0.44).

**Table 2:** Pain scores expressed as mean values and standard deviations (SD) and compared as mean difference with 95% confidence interval (CI) and *p* value (Wilcoxon matched–pairs signed–rank test).

Injection method	Sequential injections (*n* = 41)			Mixed injection (*n* = 41)			Comparison		
Parameter	Mean	SD	Range	Mean	SD	Range	Mean difference	95% CI	*P*
Pain scores	3.07	2.55	0–9	3.32	2.59	0–10	−0.24	−1.37–0.89	0.44

**Table 3:** Reported pain and preferences between the sequential and mixed injection methods, expressed as proportions (%) and compared as risk ratios with 95% confidence intervals (CI), and the Chi square test (Mantel Heinszel 2-tailed) or *Fisher's exact test.

Variables	Group frequency (*n* = 41)		Risk ratio (95% CI)	*P*
	Sequential injections	Mixed injection		
Verbal report and pain scores during MgSO_4_ injection				
No pain (pain score 0)	13/41 (32%)	12/41 (29%)	1.08 (0.56–2.08)	1
Mild pain (pain score 1–3)	12/41 (29%)	14/41 (34%)	0.85 (0.45–1.62)	0.81
Moderate pain (pain score 4–6)	13/41 (32%)	6/41 (15%)	2.17 (0.91–5.14)	0.11
Severe pain (pain score 7–10)	3/41 (7.3%)	9/41 (22%)	0.33 (0.09–1.14)	0.12
Preferred method (no preference = 6)	21/35 (60%)	14/35 (40%)	1.50 (0.92–2.44)	0.10
More painful method (no difference = 6)	13/35 (37%)	22/35 (63%)	0.59 (0.36–0.97)	0.03

Severity of pain based on the numeric pain rating scale (NPRS) (that is no pain, mild pain, moderate pain and severe pain) correlated exactly with patient verbal report of pain severity.

Severe pain was reported by 3 (7%) for the sequential injection method compared with 9 (22%) for the mixed injection method (*p* = 0.12). Six participants (15%) indicated that the perceived pain for both injection methods were similar. Of 35 who noted a difference, 13 (37%) participants rated the sequential injection method more painful whereas 22 (63%) participants rated the mixed injection method to be the more painful method (RR 0.59, 95%CI 0.36–0.97; *p* = 0.03).

Six (15%) participants did not have a preference regarding the method for future injections. Of the 35 who had a preference, 21 (60%) preferred the sequential injection method, whereas 14 (40%) preferred the mixed injection method (RR 1.50, 95% CI 0.92–2.44; *p* = 0.10).

## Discussion

This preliminary randomised controlled crossover trial aimed to evaluate the potential of a novel method of administering local anaesthesia prior to IM MgSO_4_ injection to reduce pain at the injection site compared to the current standard mixed lignocaine and MgSO_4_ method, and the participants' preference for the injection method. The findings of the study consistently favour the novel sequential injection method though the differences for most outcomes were not statistically significant in the numbers studied.

Given the widespread use of IM MgSO_4_ and the severe pain associated with this injection, it is surprising how little robust research there is on IM MgSO_4_ pain reduction with lignocaine. A randomised trial by Swathi and colleagues found that addition of 1 ml of 2% lignocaine with 10 ml of MgSO_4_ does not reduce pain at the IM injection site (5). In the same study (5), the mean pain score within five minutes of injection were somewhat higher than in our study: 5.23 for MgSO_4_ mixed with lignocaine and 5.26 for MgSO_4_ alone. It is not surprising that there is little data supporting a combined injection. Lignocaine as a local anaesthetic blocks voltage-gated sodium channels leading to a reversible block of action potential propagation. Lignocaine binds preferably to the open or inactivated state of voltage-gated sodium channels and has a rapid onset of action of 3–5 min (11, 12). In the single injection method there is presumably pain with the initial administration of the large amount of MgSO_4_ because the lignocaine has not had time to take effect. In the sequential injection method, administration of lignocaine prior to MgSO_4_ injection would presumably ease the pain of the IM injection and have sustained anaesthetic effect. The findings of the current small exploratory study suggest the potential for clinically meaningful benefit from the novel method investigated and justify further larger trials to determine the effectiveness of this method with greater precision.

The importance of attention to pain experienced by pregnant women has been highlighted by the 2018 WHO guidelines for intrapartum care for a positive birth experience, supported by an explicit requirement to document attention to pain relief measures in the new WHO labour care guide (13).

The strengths of the study are the use of a randomized crossover design with participants acting as their own controls. The limitations of the study are the small sample size and the lack of blinding. While the study could have been blinded by using a placebo initial injection in the mixed injection group, it was felt that this would obscure a potential benefit of the mixed method (only one injection) and bias the responses against the mixed injection method. Leaving the needle *in situ* for the sequential injection method may have impacted the participants' anxiety level and perception of pain. Because of the small sample pilot study, it was not possible to assess whether the amount of time between the quick succession for the loading dose participants and having four hours between the maintenance dose injections for the maintenance dose participants impacted the perception of pain, but this could be evaluated in a larger study. Additionally a larger future study may also offer the opportunity to blind using a placebo injection first in the mixed injection group, stratify the results by those receiving immediate MGSO_4_ loading dose injection vs. maintenance dosing, using blinded adjudicators to administer pain questions and ensuring accurate pain reporting training.

## Conclusion

To our knowledge this is the first study to investigate whether pre-emptive local anaesthetic injection has the potential to reduce the pain experienced by participants receiving intramuscular MgSO_4_, compared with mixing the local analgesic with the MgSO_4_. The results consistently favoured the sequential injection method. While most differences were not statistically significant in the numbers studied, the point estimate differences between groups were sufficiently large to justify a larger study to definitively determine the effectiveness of pre-emptive analgesia for IM MgSO_4_ with greater precision.

## Data Availability

The original contributions presented in the study are included in the article/**Supplementary Material,** further inquiries can be directed to the corresponding author.
