# Supplementary material for: Novel use of local analgesia prior to intramuscular magnesium sulphate injection compared to mixed local analgesia with magnesium sulphate to reduce pain: a randomised crossover study in patients being managed for eclampsia and preeclampsia
Source: Front Pain Res (Lausanne). 2024 Jul 11;5:1376608. doi: 10.3389/fpain.2024.1376608 (PMC11269267; doi:10.3389/fpain.2024.1376608)
Supplement: Supplementary file 1 [file Datasheet1.pdf]

CONSORT
TRANSPARENT REPORTING of TRIALS

CONSORT Flow Diagram

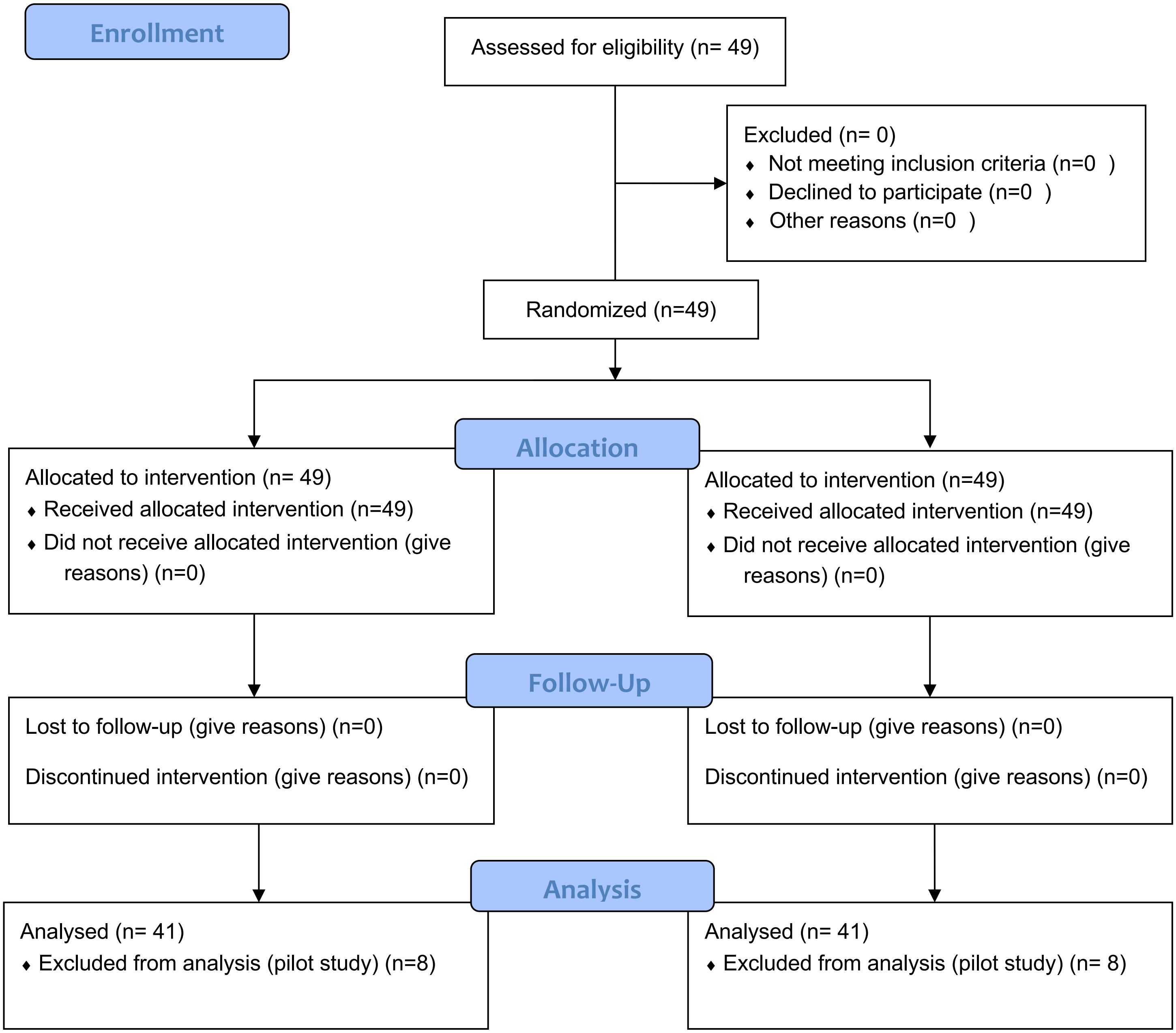

The researcher adhered to the study protocol.
